# Clinical significance of the serum miR-455-5p expression in patients with neonatal sepsis

**DOI:** 10.1080/21655979.2021.1955580

**Published:** 2021-07-21

**Authors:** Yue-Yan Mao, Chang Su, Cheng-Chao Fang, Xiao-Ping Fan, Li-Ping Wang, Shan-Shan Zhu, Hai-Ming Yao

**Affiliations:** Department of Pediatrics, The First People’s Hospital of Yuhang, Zhejiang, Hangzhou, China

**Keywords:** Neonatal sepsis, miR-455-5p, diagnosis, prognosis

## Abstract

Sepsis is a systemic inflammatory response caused by infection and is a major cause of neonatal death. This study explored the miR-455-5p in neonatal sepsis, and further investigated the diagnostic and prognostic value of miR-455-5p in neonatal sepsis (NS). The levels of serum miR-455-5p in 88 healthy controls and 90 NS patients were examined by quantitative real-time polymerase chain reaction (qRT-PCR). Pearson correlation coefficient was used to evaluate the correlation between miR-455-5p and clinical features. Receiver operating characteristic (ROC) curve analysis was performed for the diagnostic evaluation on miR-455-5p. The prognostic value of miR-455-5p in NS was analyzed by Kaplan–Meier survival curve and multivariate Cox regression. The expression of serum miR-455-5p in NS patients was highly expressed in comparison to healthy controls (*P* < 0.001), and the level of miR-455-5p was positively correlated with white blood cell count (WBC) and other clinical characteristics (*P* < 0.01). The AUC value of ROC curve was 0.895, suggesting that miR-455-5p had diagnostic value for NS. Survival analysis illustrated that patient with high miR-455-5p expression had poor prognosis (log rank *P* = 0.015), and miR-455-5p may be a potential prognostic marker for NS (HR = 3.454, 95% CI = 1.165–10.234, *P* = 0.025). The expression of miR-455-5p had the ability to distinguish NS from healthy people, and highly expressed miR-455-5p was associated with poor prognosis in NS patients.

## GRAPHICAL ABSTRACT

**Figure:**
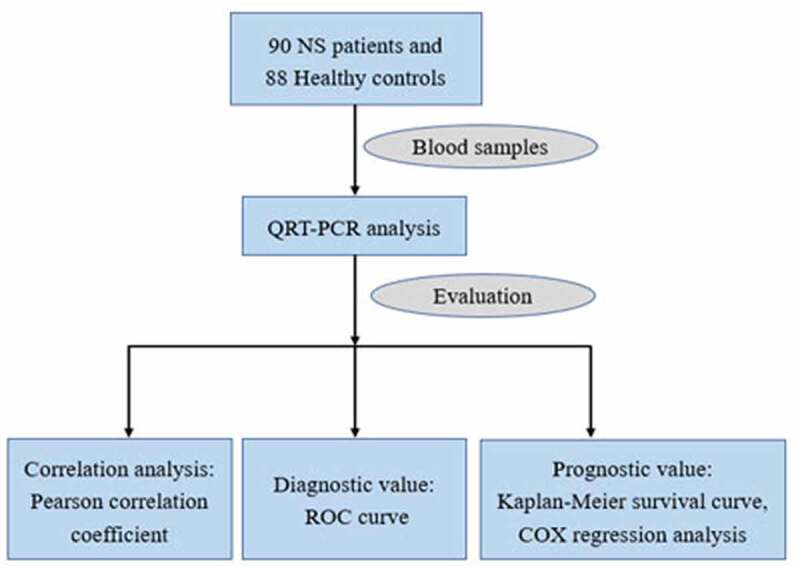

## Introduction

Sepsis is considered a systemic inflammatory response syndrome caused by infection of pathogenic microorganisms, including bacteria, fungi and viruses [1,2]. Due to the weak constitution and the imperfect development of the immune system, neonates are more prone to infection and sepsis, which may lead to shock or even death in severe cases [3,4]. In the early stages of sepsis, neonatal sepsis (NS) patients do not show obvious symptoms, and the presence of symptoms often indicated that the disease has been severe enough [5]. Blood culture was once considered as the gold standard for the diagnosis of sepsis, but its long diagnostic cycle and susceptibility to false negative results make it extremely detrimental for severe patients, especially newborn patients [6]. In the process of disease development, it is one of the effective means to delay the development of disease to make timely judgment and carry out targeted intervention. Therefore, the exploration of new biomarkers for sepsis has special clinical significance.

Currently, biomarkers with poor sensitivity and specificity examined in the laboratory, including C-reactive protein (CRP), Procalcitonin (PCT) and neutrophil–lymphocyte ratio (NLR) are limited in diagnosis [7,8]. In recent decades, a great number of studies have focused on the analysis of circulating microRNA (miRNA). In essence, miRNAs are a new group of small RNAs that regulate gene expression but do not encode proteins [9]. MiRNAs have been described during the growth, development, and senescence of the body, but they are also involved in the occurrence of complex diseases, such as tumor, autoimmune disorders, infectious diseases [10,11]. Yao et al. demonstrated that miR-25 was more accurate in the diagnosis of sepsis than established markers such as CRP and PCT, and they also found that miR-25 levels were associated with poor outcomes in sepsis patients [12]. Nong et al. showed that the overexpression of miR-126 prevented brain damage in sepsis rats by inhibiting the NF-κB signaling pathway [13]. Huang et al. identified miRNA biomarkers for pneumonia by RNA sequencing and bioinformatics analysis, and they found that the plasma expression of miR-455-5p was significantly higher in patients with severe pneumonia than in healthy patients [14]. Laura et al. demonstrated that miR-455 was highly expressed in the lipopolysaccharide (LPS) induced rat model of inflammation and was involved in the regulation of the immune response [15]. Multiple evidence showed that miR-455 was related to the regulation of inflammatory diseases and immune function. Considering that the pathological manifestations of sepsis are involved in inflammatory response, there is no relevant study on miR-455 and sepsis at present. Therefore, the correlation between miR-455-5p and sepsis remains to be further investigated.

Based on the above results, we speculated that miR-455-5p may play a certain role in NS. To study the role and clinical significance of miR-455-5p in NS, we analyzed the expression of miR-455-5p in the serum of NS patients and evaluated the diagnostic and prognostic value of miR-455-5p in NS using an ROC curve and a 28-day follow-up study. Our study showed that miR-455-5p had diagnostic value for NS, and patients with high expression of miR-455-5p had poor prognosis. In general, our study provided a new perspective for the diagnosis and prognosis of NS, and it also laid a theoretical foundation for revealing the clinical role of miR-455-5p in NS.

## Material and methods

### Study population and sample collection

This research was conducted in the Neonatal Intensive Care Unit (NICU) of the First People’s Hospital of Yuhang from January 2019 to August 2020. This study was carried out after obtaining the approval of the ethics committee of the First People’s Hospital of Yuhang. The guardians of all newborns were informed of this study and signed the written informed consent.

A total of 178 neonates were recruited in this study, including 90 neonates diagnosed with sepsis and 88 healthy neonates as controls. The sample size was estimated according to preliminary data. α = 0.05, β = 0.1, the power = 0.9 and a drop-out rate of 10%, at least 85 cases in each group were tested according to the two-tailed test. Therefore, the sample size of this study met the requirements. The diagnosis of NS is based on the neonates’ clinical manifestations, medical history, and laboratory test results [16,17]. Clinical manifestations meeting three or more of the following symptoms can be determined. 1) Hyperthermia or hypothermia, 2) Cardiac dysfunction, including tachycardia, bradycardia, and blood pressure reduction, 3) Neurological disorders, occurrence of drowsiness, convulsions, epilepsy, 4) apnea, tachypnea, cyanosis, 5) Gastrointestinal disorders, such as diarrhea and abdominal distension. Eighty-eight healthy newborns who were born in this hospital during the same period were selected as the control group. Exclusion criteria: 1) premature neonates, 2) congenital malformation or chromosomal abnormality. Blood samples from NS patients and the control group were collected for various biochemical tests before systematic treatment.

### RNA extraction and qRT-PCR analysis

The expression level of miR-455-5p was detected by qRT-PCR and the procedure for qRT‐PCR quantification was described in previous reports [18]. Briefly, total RNA was extracted by adding Trizol reagent to subjects’ serum samples, and the RNA was reverse transcribed into cDNA using a reverse transcription kit. According to the instructions, the Miscript SYBR Green PCR Kit was used on the ABI 7300 real-time PCR system, and U6 was considered as the internal reference in the reaction. Finally, the relative expression level of miR-455-5p was calculated according to the experimental results and 2^−ΔΔCt^ method. The primers used in this study are as follows: miR-455-5p, forward: 5ʹ-GTATGTGCCTTTGGACTACAT-3ʹand reverse: 5ʹ-GTCGTATCCAGTGCAGGG-3ʹ. U6, forward: 5ʹ-CTCGCTTCGGCAGCACA-3ʹ and reverse: 5ʹ-AACGCTTCACGAATTTGCGT-3ʹ. qRT-PCR amplification conditions were as follows: 1 cycle of 95°C for 5 minutes (pre-denaturation), followed by 40 cycles of 95°C for 10 seconds (denaturation), 60°C for 20 seconds (annealing) and 72°C for 15 seconds (extension).

### 28-day follow-up analysis

Ninety NS patients were enrolled in a 28-day follow-up study after treatment, and they were divided into two groups according to the cutoff value (1.51), which was defined as the median value of the miR-455-5p expression as follows: high miR-455-5p expression group (n = 51) and low miR-455-5p expression group (n = 39). After the follow-up, the Kaplan–Meier survival curve was drawn based on the survival rate of the neonates according to previous studies [19].

### Data analysis

Data analysis was performed using SPSS 21.0 and GraphPad Prism 7.0. The normality of experimental data was analyzed by Kolmogorov–Smirnov (K-S) normality test. Except for the data of age, the data conforming to the normal distribution were represented as mean ± SD. The differences between groups were estimated by student t test and one-way ANOVA. The comparison between qualitative variables was verified by the chi-square test. Pearson correlation coefficient analysis was implemented for the study of correlation between miR-455-5p and clinical characteristics. The diagnostic value of miR-455-5p was assessed by an ROC curve, and the prognostic value of miR-455-5p was evaluated by Kaplan–Meier method and multivariate Cox regression analysis. *P* < 0.05 was considered as a significant difference.

## Results

### Baseline information of subjects

In the present study, 90 NS patients and 88 healthy neonates were included. Demographic characteristics and clinical data of the two groups are shown in Table 1. WBC, CRP, PCT and NLR in NS patient group were generally higher than that in healthy controls (*P* < 0.001), while there was no statistical difference in age, body weight, gender, and other indicators between the two groups (*P* > 0.05).

**Table 1.** Clinical data of the study population

Parameters	Subjects (N = 178)		*P*
	Healthy individuals (n = 88)	Neonatal sepsis (n = 90)	
Age (days)	11.41 ± 5.10	10.21 ± 4.04	0.084
Body weight (kg)	3.39 ± 0.53	3.40 ± 0.46	0.976
Gender (male/female)	37/51	43/47	0.442
WBC (×10^9^/L)	8.02 ± 3.10	17.51 ± 6.04	<0.001
CRP (mg/L)	4.87 ± 3.27	15.20 ± 4.30	<0.001
PCT (ng/mL)	0.17 ± 0.05	10.69 ± 5.11	<0.001
NLR	1.22 ± 0.3	5.38 ± 2.21	<0.001

WBC, white blood cell; CRP, C-reactive protein; PCT, procalcitonin; NLR, neutrophil–lymphocyte ratio. Data are expression mean ± standard deviation.

### Expression level of miR-455-5p in NS patients

**Figure 1.**
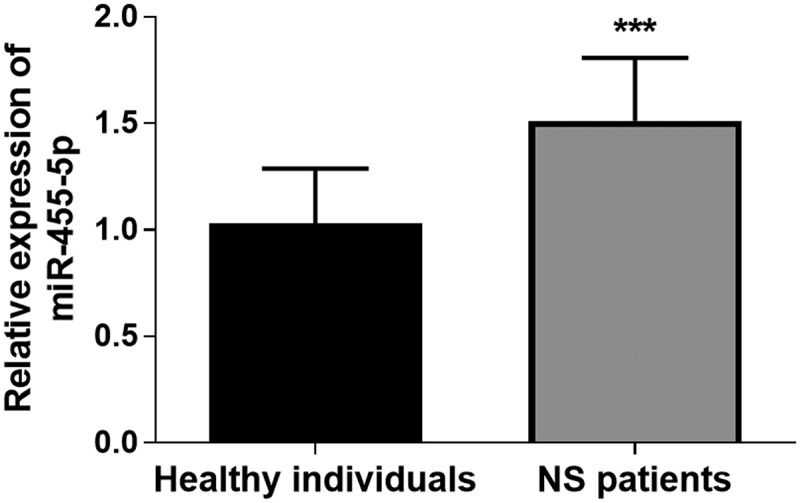
The relative expression of miR-455-5p in NS patients. *** *P* < 0.001

To investigate whether the expression of miR-455-5p was abnormal in the serum of all subjects, the expression levels of miR-455-5p in blood samples of all subjects were analyzed by qRT-PCR technology. As illustrated in Figure 1, it was found that the expression of serum miR-455-5p in NS patients was significantly increased in comparison to healthy controls (*P* < 0.001). The abnormal expression of miR-455-5p suggested that this miRNA might be involved in the regulation of NS.

### Pearson correlation analysis and ROC curve analysis

**Figure 2.**
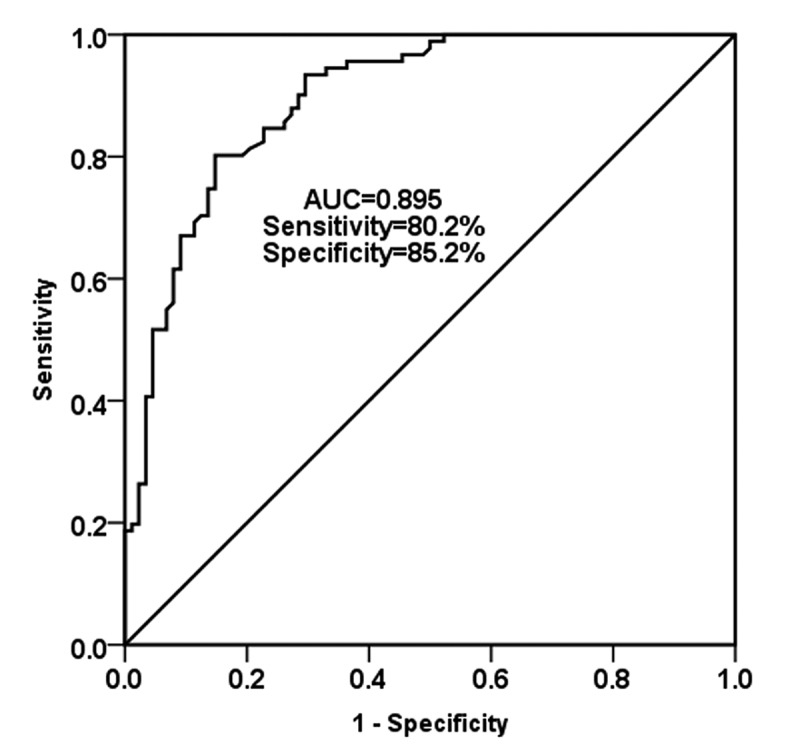
The diagnostic value of miR-455-5p was assessed by the ROC curve analysis. the AUC was 0.895, with the sensitivity of 80.2% and the specificity of 85.2%

Considering the abnormal expression of miR-455-5p in NS and the significance of miR-455-5p level in the course of the disease, we evaluated the correlation between miR-455-5p level and patients’ clinical indicators through Pearson correlation coefficient. Meanwhile, in order to investigate whether miR-455-5p has clinical diagnostic value in NS, the ROC curve was generated and the area under the curve (AUC) value was calculated. In Table 2, it was found that the level of miR-455-5p was significantly positively correlated with the level of WBC, CRP, PCT, NLR, IL-6, IL-8 and TNF-α (*P* < 0.01), which conveyed by this result is that the overexpression of miR-455-5p may contribute to the progression of NS. Furthermore, the ROC curve evaluated the diagnostic value of miR-455-5p in NS. As shown in Figure 2, we found that the AUC was 0.895, accompanied by a sensitivity of 80.2% and specificity of 85.2% at the cutoff value of 1.2535, which indicated that miR-455-5p has high diagnostic value in NS patients.

**Table 2.** Correlation between miR-455-5p and clinical characteristics

Characteristics	N	Correlation with miR-455-5p (r)	*P-value*
WBC (×10^9^/L)	90	0.382	0.001
CRP (mg/L)	90	0.392	<0.001
PCT (ng/mL)	90	0.308	0.003
NLR	90	0.346	0.001
IL-6 (pg/mL)	90	0.360	<0.001
IL-8 (pg/mL)	90	0.308	0.003
TNF-α (pg/mL)	90	0.486	<0.001

WBC, white blood cell; CRP, C-reactive protein; PCT, procalcitonin; IL-6, interleukin-6; IL-8, interleukin – 8; TNF-α, tumor necrosis factor -alpha; NLR, neutrophil – lymphocyte ratio.

### Analysis of the prognostic significance of miR-455-5p in NS

**Figure 3.**
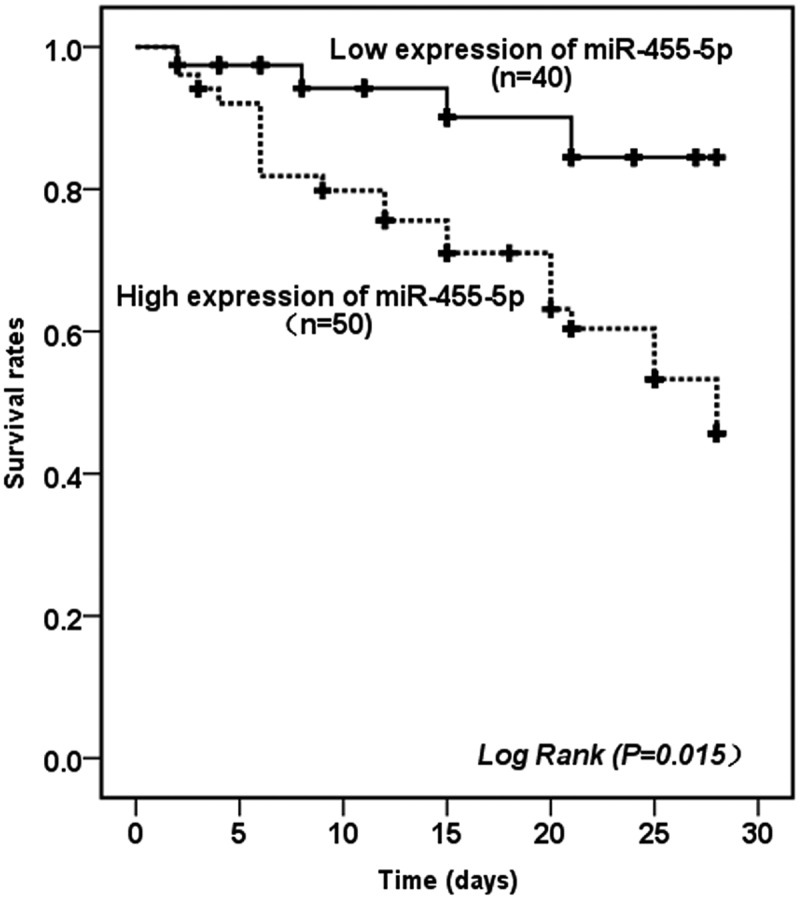
Kaplan-Meier survival curves of NS patients with different miR-455-5p expression levels

To further evaluate the prognostic value of miR-455-5p for NS patients, 90 NS patients were enrolled in a 28-day follow-up trial, and a Kaplan–Meier survival based on the survival rate at the specified time was established. Among the 90 neonates who participated in the follow-up, 4 patients in the low miR-455-5p expression group died within 28 days, while the number of deaths in the high miR-455-5p expression group was as high as 21 cases. The Kaplan–Meier survival curve is shown in Figure 3. The results revealed that the probability of poor prognosis was significantly higher in patients with high miR-455-5p expression than in patients with low miR-455-5p expression (log-rank *P* = 0.015). Besides, to further evaluate the prognostic value of miR-455-5p in NS, we performed multivariate COX regression analysis on relevant data. The results in Table 3 shows that highly expressed of miR-455-5p was an independent predictor of death within 28 days in NS patients (HR = 3.454, 95% CI = 1.165 − 10.234, *P* = 0.025).

**Table 3.** Multivariate Cox analysis of clinical characteristics in relation to overall survival

Characteristics	Multivariate analysis		
	HR	95% CI	*P*
MiR-455-5p	3.454	1.165–10.234	0.025
Age (days)	1.430	0.601–3.405	0.419
Body weight (kg)	1.252	0.547–2.867	0.595
Gender (male/female)	1.399	0.596–3.283	0.441
WBC (×10^9^/L)	2.021	0.850–4.806	0.112
CRP (mg/L)	1.411	0.620–3.212	0.411
PCT (ng/mL)	2.432	1.000–5.914	0.050
NLR	1.464	0.642–3.336	0.365

Abbreviations: WBC, white blood cell; CRP, C-reactive protein; PCT, procalcitonin; NLR, neutrophil–lymphocyte ratio.

## Discussion

Sepsis is a very serious state in neonatal infectious diseases [20]. Neonates may eventually develop multiple-organ dysfunction syndrome with poor prognosis as the disease progresses [16]. With the continuous development of medical technology, although there has been a comprehensive and systematic treatment program for the sepsis treatment, the mortality rate of sepsis is still high due to the particularity of neonates [21]. In a word, it is extremely urgent to find biomarkers with high sensitivity and specificity to deal with NS.

As previous studies have pointed out, researchers have detected miRNAs in various body fluids, including sweat, blood, urine, and saliva [1,22]. miR-455 family, located in the fragile site region of chromosome 9q32, is a tumor-related miRNA molecule, which is abnormal expressed in a variety of tumors, including, gastric cancer, breast cancer, colorectal cancer [23–25]. For example, as a tumor suppressor gene, miR-455-5p inhibited the proliferation and metastasis of gastric cancer cells by down-regulating the expression of Rab18 [26]. Nowadays, more and more evidence has confirmed that miR-455-5p was participated in the regulation of inflammatory response and infection diseases with the in-depth study of this gene. Vinay et al. pointed out that miR-455 was expressed highly in intestinal epithelial dysfunction caused by HIV infection [27]. Cui et al. confirmed the high expression of miR-455-5p in the serum of children with enterovirus infection in foot-and-mouth disease [28]. In addition, Travis et al. found that inflammatory miRNAs such as miR-455 and miR-142 were overexpressed in a mouse model of severe skeletal muscle inflammation [29]. The results of our study are consistent with previous findings on miR-455 in infectious diseases, and our study confirmed that miR-455-5p expression was enhanced in the serum of NS patients. Meanwhile, Pearson correlation coefficient analysis exhibited that the level of miR-455-5p in NS patients was significantly positively correlated with the levels of WBC, CRP, PCT, NLR, and the levels of inflammatory factors, including IL-6, IL-8, TNF-α, We speculated that the expression level of miR-455-5p was closely related to the degree of inflammatory response in sepsis.

Inflammation is the most obvious feature in the pathological process of NS. After infection, a great number of neutrophils stored in the bone marrow were released into the blood, while the specific immune activation and injury of lymphocytes resulted in the decrease of lymphocytes [30]. As a result, the ratio of neutrophils to lymphocytes (NLR) gradually increased with the aggravation of the inflammatory response [31]. CRP, an acute-phase protein, was produced by the liver in response to inflammation and/or infectious stimuli [32]. When the body is infected, the level of CRP in the blood increased sharply. However, the increased CRP was often seen in other conditions, such as stress response and non-infective state [33]. Due to the lower specificity, CRP is not suitable as a single marker for diagnosis of disease. Serum concentrations of PCT are extremely low in healthy people, while PCT was synthesized and released into the blood in large quantities only in the event of a severe systemic infection [34]. To some extent, PCT is an indicator of the severity of infection. Study reported that the specificity of PCT in the diagnosis of NS was 79% [35]. Although PCT seems to be more qualified as a diagnostic marker for NS, some studies also suggested that PCT was suitable for guidance and management of NS and should not be used in the diagnosis of NS alone [36]. Combined with our findings, we have reason to confirm that the high expression of miR-455-5p was undoubtedly positively correlated with the severity of sepsis. Furthermore, the high sensitivity and specificity of the ROC curve proved the diagnostic value of miR-455-5p in NS. Therefore, the high expression of miR-455-5p was considered as a new potential and effective biomarker for NS diagnosis in this study. In this study, NS patients participated in a 28-day follow-up study with neonatal death as the endpoint event. It was observed that the probability of death in NS patients with high miR-455-5p expression was higher than that in NS patients with low miR-455-5p expression. Meanwhile, multivariate COX regression analysis illustrated that miR-455-5p expression was an independent prognostic factor for NS. The above results revealed that miR-455-5p has high diagnostic and prognostic value in NS.

Like other experimental studies, this study also has some limitations that should not be ignored. On the one hand, this study only evaluated the diagnostic and prognostic value of miR-455-5p for NS, and we did not investigate the specific mechanism and biological function of miR-455-5p in NS. Besides, we need to explore the function of miR-455-5p in animal models, and more experiments are needed to verify the molecular mechanisms of miR-455-5p. On the other hand, we were unable to expand the sample size to evaluate the expression of miR-455-5p. Therefore, it is uncertain whether data consistent with the current results can be obtained after enlarging the sample size. In future studies, we will design more rigorous experimental plans to verify the role and mechanism of miR-455-5p as a biomarker in the development of NS.

## Conclusion

In summary, this study proved that miR-455-5p was highly expressed in NS patients through related experiments. The up-regulated miR-455-5p indicated the occurrence of NS, and similarly, the highly expressed miR-455-5p also indicated poor prognosis of NS patients. Based on the above results, we believe that miR-455-5p has the potential to be a biomarker for the diagnosis and prognosis of NS. Due to the lack of experimental data, experimental results need to be continuously reevaluated and clinically validated.

## Data Availability

The data that support the findings of this study are available from the corresponding author upon reasonable request.

## Highlights

1. The serum expression of miR-455-5p was elevated in neonatal sepsis (NS) patients.
2. miR-455-5p had the ability to distinguish NS patients from healthy controls.
3. Patients with high expression of serum miR-455-5p have a poor prognosis in NS.
